# Pointwise prediction of protein diffusive properties using machine learning

**DOI:** 10.1088/2515-7647/adede9

**Published:** 2025-07-17

**Authors:** Rasched Haidari, Achillefs N Kapanidis

**Affiliations:** 1 Gene Machines Group, Clarendon Laboratory, Department of Physics, University of Oxford, Oxford, United Kingdom; 2 Kavli Institute of Nanoscience Discovery, Dorothy Crowfoot Hodgkin Building, University of Oxford, Oxford, United Kingdom

**Keywords:** anomalous diffusion, pointwise inference, diffusion, AnDi_2_ challenge, machine learning, LSTM, changepoint analysis

## Abstract

The understanding of cellular mechanisms benefits substantially from accurate determination of protein diffusive properties. Prior work in this field primarily focuses on traditional methods, such as mean square displacements, for calculation of protein diffusion coefficients and biological states. This proves difficult and error-prone for proteins undergoing heterogeneous behaviour, particularly in complex environments, limiting the exploration of new biological behaviours. The importance of determining protein diffusion coefficients, anomalous exponents, and biological behaviours led to the Anomalous Diffusion Challenge 2024, exploring machine learning methods to infer these variables in heterogeneous trajectories with time-dependent changepoints. In response to the challenge, we present M3, a machine learning method for pointwise inference of diffusive coefficients, anomalous exponents, and states along noisy heterogenous protein trajectories. M3 makes use of long short-term memory cells to achieve small mean absolute errors for the diffusion coefficient and anomalous exponent alongside high state accuracies (>90%). Subsequently, we implement changepoint detection to determine timepoints at which protein behaviour changes. M3 removes the need for expert fine-tuning required in most conventional statistical methods while being computationally inexpensive to train. The model finished in the Top 5 of the Anomalous Diffusive Challenge 2024, with small improvements made since challenge closure.

## 1 Introduction

Protein diffusion is vital to understanding cellular processes and the mechanisms which govern cellular functionality [1–4]. Advancements in fields such as single-molecule imaging and single-particle tracking have allowed for direct experimental observation of protein movement [4–15]. These studies have made extensive use of spatial-temporal properties of protein trajectories such as mean square displacements (MSD) and diffusion coefficients. Subsequent hard thresholding of diffusion coefficients allows for categorisation of proteins into different states, with each state representing underlying biological behaviour (e.g. a RNA polymerase molecule with a low diffusion coefficient may be interacting with the DNA) [14, 16–18].

Although successful for a small number of states with relatively simple behaviours, MSD approaches are difficult to extend to complex behaviours such as changes in environment (e.g. viscosity), confinement or directed motion [19–21]. This is further complicated with proteins transitioning between different states, leading to inaccurate diffusion coefficients and overlapping states causing misclassifications [22].

To address these issues, Muñoz–Gil *et al* introduced the Anomalous Diffusion (AnDi) Challenge in 2020 [21]. This challenge focused on advanced statistical and machine learning approaches to predict anomalous exponents and diffusion type for protein trajectories undergoing at most a single changepoint between states. The anomalous exponent is essential in understanding protein movement and is related to the diffusion coefficient by the equation [23, 24]:

\begin{equation*}{\text{MSD}}\left( t \right) = 4K{t^\alpha }\end{equation*}

where $K$ is the diffusion coefficient and *α* is the anomalous exponent. There are three distinct cases of α for which changes in diffusive behaviour are observed:

• $a &lt; 1$ —sub-diffusion, protein trajectories display ‘confined’ steps; ${\text{MSD}} \propto {t^\alpha }$ ($\alpha = 0$ representing immobile motion)
• $a$ = 1 corresponds to Brownian motion; ${\text{MSD}} \propto t$
• $1\, &lt; a &lt; 2$ —super-diffusion, protein trajectories display ‘directed’ steps; ${\text{MSD}} \propto {t^\alpha }{ }$ ($\alpha = 2$ representing ballistic motion)

The above MSD formula can be used to describe fractional Brownian motion (fBM), a generalisation of Brownian motion, in which the increments in position are no longer independent [23–28]. The covariance function for FBM is given by [24]:

\begin{equation*}{\text{Cov}}\left[ {{B_H}\left( t \right){B_H}\left( s \right)} \right] = K\left( {{{\left| t \right|}^{2H}} + {{\left| s \right|}^{2H}} - {{\left| {t - s} \right|}^{2H}}} \right)\end{equation*}

where ${B_H}$ is the stochastic process, $t$ and $s$ are time points, and $H$ is the Hurst exponent given by $\alpha = \,2H$ [24]. When $\alpha \, \ne 1\,$, different timesteps of the protein trajectories are negatively or positively correlated, leading to ‘confined’ or ‘directed’ behaviour respectively.

The outcome of AnDi 2020 led to more accurate machine learning techniques as compared to traditional MSD methods [21]. Machine learning models, developed both during and outside of the challenge, have implemented various architectures such as recurrent neural networks, convolutional neural networks, graph neural networks, feed-forward networks with feature engineering, and more recently transformers [29–45].

The success of the first challenge led to AnDi 2024 [46], in which the problem focused more specifically on fBM of proteins undergoing different biological behaviours and an arbitrary number of changepoints between these states. Given noisy protein coordinates, $x\left( t \right)$ and $y\left( t \right)$, the task is to infer $K$, $\alpha $, and the protein state ($s$) over the timeseries, where each variable can have an unknown number of arbitrary changepoints.

### 1.1 AnDi challenge 2024

**Figure 1.**
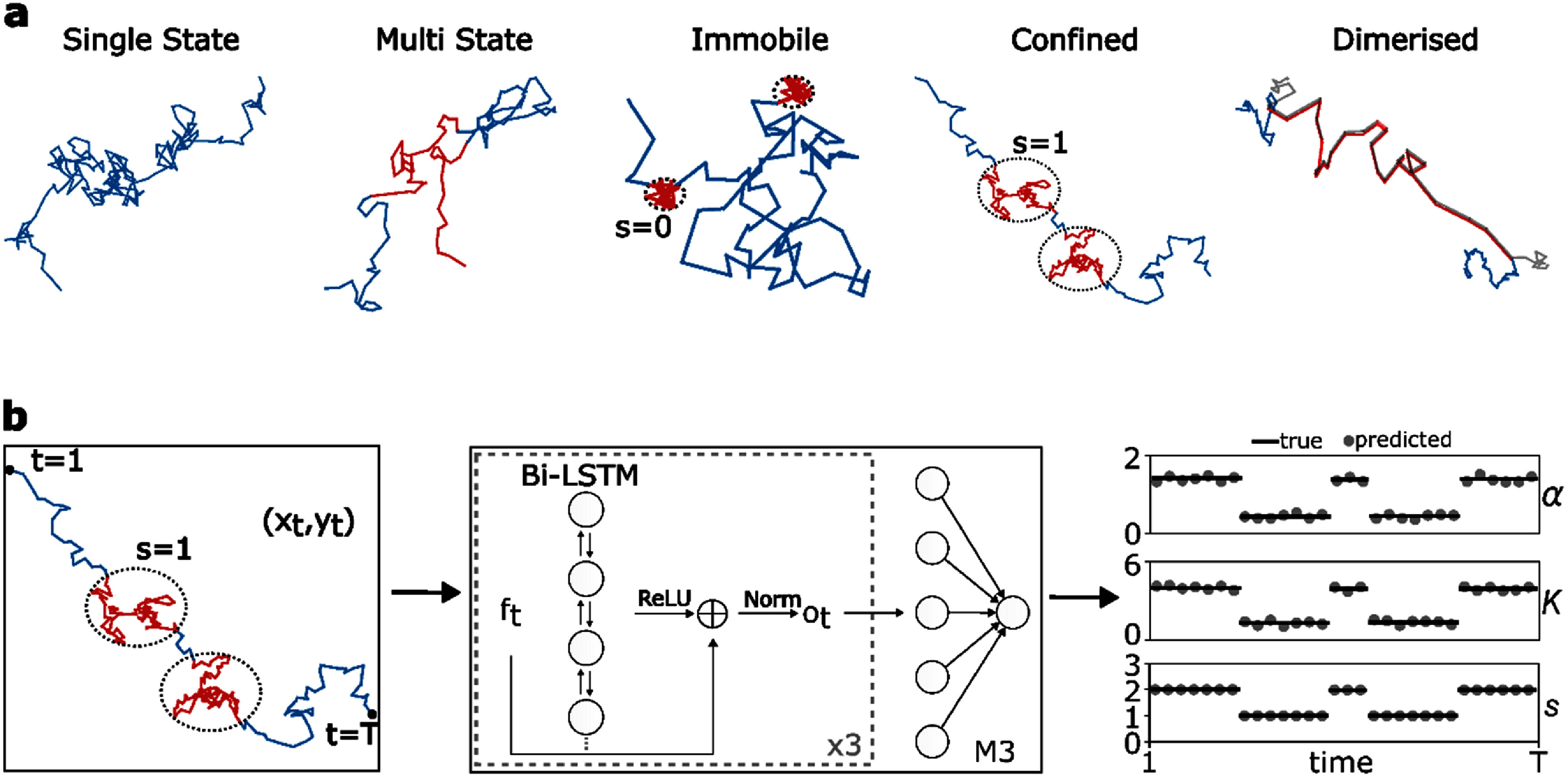
(a) Biological behaviours simulated using the AnDi package [49]. Red paths denote a change in behaviour. Immobile regions are assigned a state, $s$, equal to zero, while confined regions are assigned a state value of one. Unless specified, the state is either two or three. Figure Reproduced from [46]. CC BY 4.0. (b) M3 model architecture. The model takes in a protein trajectory and extract features *f*_t_. This is passed through a bidirectional LSTM and we concatenate with the input using skip connections and subsequently layer normalise. This is performed three times, followed by a feed-forward layer providing pointwise predictions. The model is retrained for every variable.

AnDi 2024 considers protein trajectories simulated from five different biological behaviours as shown in figure 1(a). A single state model (SSM) does not have any changepoints, providing a control for the sensitivity of the model in the detection of changes. The rest of the models can exhibit an arbitrary number of changepoints either between two or more states as given by a transition matrix. At these changepoints, at least one of $K$, $\alpha $ or $s$ will change. The multi-state model (MSM) considers time-dependent changes; the quenched-trap model (QTM) considers proteins in trapped (immobile) regions; the transient-confinement model considers proteins entering and exiting compartments; and lastly the dimerization model (DIM) considers co-diffusion between interacting proteins. The model can be extended to higher-order oligomeric states, which would involve scanning localisation coordinates for particles within Gaussian noise of each other. This would not require any further changes to the model (rather just data filtering) and such particles can be assigned into groups.

For QTM, $K = \,\alpha = s = \,0$ when the protein is trapped. For the rest of the diffusive states, we have $\alpha \, \in \,\left[ {0,2} \right],\,\,K \in \,\left[ {{{10}^{ - 12}},\,{{10}^6}} \right]$, and $s\, \in \left\{ {0,1,2,3} \right\}$. Note there are five underlying biological behaviours but four states. These represent immobile (imm.) motion ($s = 0$ with $\alpha = 0$), confinement (conf.) ($s = 1$ with $0\, &lt; \alpha &lt; 1.9$), free diffusion ($s = 2$ with $0\, &lt; \alpha &lt; 1.9$), and directed (dir.) motion ($s = 3$ with $\alpha &gt; 1.9$) [46]. The value of $\alpha $ or $K$ does not necessarily have to change with $s$.

In response to the AnDi 2024 challenge, we present M3, a long short-term memory (LSTM) based method for the pointwise inference of $K$, $\alpha $, $s$, and subsequent changepoint detection using the Python package ruptures [47, 48]. M3, as depicted in figure 1(b), extracts multivariate time series features from the original coordinates of the protein trajectory and passes these into bidirectional LSTM (biLSTMs) layers (with skip connections and dropout), followed by a feedforward layer. The use of LSTMs with skip connections allows the model to pick up on long-range dependencies while keeping the architecture simple.

## 2 Methods

### 2.1 Model architecture

As we are dealing with timeseries data, we implement a custom sequence-to-sequence model based on LSTM cells. Notably, we have three stacked biLSTMs with Rectified Linear activation functions, skip connections, and layer normalisations (see figure 1(b)). The first two biLSTM cells have two hidden layers and the last biLSTM has a single layer, with all three cells returning a vector of length 128. The output of the last biLSTM is passed onto a fully connected layer, mapping the result into the desired shape. The input to the model is ten features (see *2.3* *Feature Selection*), in the form of timeseries, extracted from the initial coordinates $x\left( t \right)$ and $y\left( t \right)$. The model returns a sequence of $K\left( t \right)$, $\alpha \left( t \right)$ or $s\left( t \right)$ for every timestep. For each of the three output variables, we duplicate the model and retrain, with the exception of adding a LogSoftmax layer before the output of the state model (classification task). M3 has a total of roughly 513 000 trainable parameters per model.

The model architecture resulted from starting with a single biLSTM cell and progressively incrementing the number of cells and layers until no improvement in validation loss was seen. The same procedure was carried out for other parameters in the model such as the dropout rate, number of fully connected layers, learning rate and regularisation parameter. To streamline this process, we use Optuna [50], an open source Python library implementing Bayesian hyperparameter search, and some trial and error (see *2.5* *Training* for hyperparameter values and *Data Availability* for tuning process).

### 2.2 Training data

Training data was simulated using the AnDi Python package [21, 46, 49]. Table 1 shows the general parameters used for model training. Briefly, we simulate 400 000 trajectories each for the single state, multi-state, immobile, confined, and dimerised models. This leads to a total of two million trajectories. We repeat this process for the case where $\alpha $ is constrained to [1.9, 2]. The constrained range for $\alpha $ simulated more trajectories displaying directed motion ($s = 3$), a minority class. We further simulated trajectories (using the same sampling ranges as in table 1) in which changepoints occurred in only one of $\alpha $ or $K$ while the other remained fixed across the changepoint. This is only possible for the multi-state, dimerised, and confined models, allowing isolation of $\alpha $ or $K$ without interference from the other. In total we obtained 5.2 million trajectories. The trajectories are split randomly into train/test/validation datasets in a ratio of 60:20:20, such that there are 3.12 million trajectories in the training data. A few figures have been generated using different datasets, but this will be described in the text. Units for $K$ are ${\text{pixel}}{{\text{s}}^2}/{\text{ fram}}{{\text{e}}^\alpha }$.

**Table 1.** Parameters for simulation using the AnDi Python package [21, 46, 49].

Model	Parameters	Sampling range
Single state (SSM)	$\alpha $ $K$	[0,2] [10^−12^, 10^6^]

Multi state (MSM)	Number of states (*N*_s_) Transition matrix *α_i_* for *i* = 1, …, *N_s_* *K_i_* for *i* = 1, …, *N_s_*	{2, 3} [0, 1]*^Ns^*^×*Ns*^ [0, 2] [10^−12^, 10^6^]

Quenched trap (QTM)	Unbinding probability Binding probability Trap radius Number of traps Free *α, K* Trapped *α, K*	[0, 0.1] 1 [0.5, 2] [100, 300] [0, 2], [10^−12^, 10^6^] 0, 0

Dimerization (DIM)	Unbinding probability Binding probability Interaction radius Free *α, K* Dimerised *α, K*	[0, 0.1] 1 [0.5, 5] [0, 2], [10^−12^, 10^6^] [0, 2], [10^−12^, 10^6^]

Transient confinement (TCM)	Transition probability Number of compartments Compartment radius Free *α, K* Confined *α, K*	[0, 0.3] [30, 50] [5, 10] [0, 2], [10^−12^, 10^6^] [0, 2], [10^−12^, 10^6^]

### 2.3 Feature selection

We carry out an extensive literature search, including past AnDi challenge literature, to obtain features specific to the problem [21, 29–46, 51–59]. We add generic timeseries features to this (e.g. running means and standard deviations) resulting in a list of roughly 80 features. Using Pearson’s correlation coefficient (PCC) we remove highly collinear features (|PCC| > 0.95). Then, we perform greedy forward feature selection starting with the *z*-normalised (zero mean and unit variance) $x\left( t \right)$ and $y\left( t \right)$. By symmetry, a feature not improving the model in $x$ was removed alongside it is $y$ counterpart (e.g. $x$ and $y$ running standard deviation).

After this process, we were left with ten features, each being a timeseries itself, derived from the original coordinates. These are:

• $z$ -normalised $x$ and $y$ coordinates $\hat x\left( t \right) = \,\frac{{x\left( t \right) - \,\bar x}}{{{\sigma _x}}}$ and $\hat y\left( t \right) = \,\frac{{y\left( t \right) - \,\bar y}}{{{\sigma _y}}}$
• displacement from origin $\,d\left( t \right) = \sqrt {{{\left( {x\left( t \right) - x\left( 0 \right)} \right)}^2} + {{\left( {y\left( t \right) - y\left( 0 \right)} \right)}^2}} $
• $z$-normalised step size $\widehat {{\text{step}}}\left( t \right) = { }\frac{{{\text{step}}\left( t \right) - { }\overline {{\text{step}}} }}{{{\sigma _{{\text{step}}}}}}$ with ${\text{step}}\left( t \right) = { }\sqrt {\Delta x{{\left( t \right)}^2} + \Delta y{{\left( t \right)}^2}} $ and $\Delta $ is the difference between consecutive elements in the timeseries.
• Angle between segments of the trajectory $\vartheta \left( t \right)$
• ${\text{str}}\left( t \right) = \frac{{\sqrt {{{\left( {\hat x\left( t \right) - \hat x\left( 0 \right)} \right)}^2} + {{\left( {\hat y\left( t \right) - \hat y\left( 0 \right)} \right)}^2}} }}{{\sum\nolimits_{j = 1}^t {\sqrt {\Delta \hat x{{\left( j \right)}^2} + \Delta \hat y{{\left( j \right)}^2}} } }}$
• ${\text{st}}{{\text{r}}_2}\left( t \right) = \frac{{{{\left( {\hat x\left( t \right) - \hat x\left( 0 \right)} \right)}^2} + {{\left( {\hat y\left( t \right) - \hat y\left( 0 \right)} \right)}^2}}}{{\sum\nolimits_{j = 1}^t \Delta \hat x{{\left( j \right)}^2} + \Delta \hat y{{\left( j \right)}^2}}}$
• $F\left( {\hat x} \right)$, $F\left( {\hat y} \right)$, and $F\left( {\widehat {{\text{step}}}} \right)$ where $F\left( k \right) = \log \left| {\Delta k\left( t \right)} \right|.$

The features ${\text{str}}\left( t \right)$ and ${\text{st}}{{\text{r}}_2}\left( t \right){ }$ are measures of trajectory straightness, where a value of 1 represents perfectly directed motion (straight path) and a value of 0 represents a path which returned to its starting position. The final input to the model is [$\hat x,\hat y,{ }d,{ }\widehat {{\text{step}}},{ }\vartheta ,{\text{ str}},{\text{ st}}{{\text{r}}_2},{F_{\hat x}},{F_{\hat y}},{ }{F_{\widehat {{\text{step}}}}}$]. Any features shorter than the total number of timesteps is post-padded with zeros. The input to the model is of shape (*B*, 10, *T*) where *T* is the maximum trajectory length in the batch and *B* is the batch size (see *2.5* *Training*).

### 2.4 Augmentations

We add the following augmentations to the coordinates $x\left( t \right)$ and $y\left( t \right)$:

• Gaussian noise with $\mu = 0$ and $\sigma = 0.1$ pixels, $y{^{^{\prime}}}\left( t \right) = y\left( t \right) + { }\varepsilon \left( t \right),{ }x{^{^{\prime}}}\left( t \right) = x\left( t \right) + { }\varepsilon \left( t \right),{ }\varepsilon \left( t \right)\sim \mathcal{N}\left( {0,0.1} \right).$
• Random rotations, $\left[ {x^{\prime}\left( t \right),y^{\prime}\left( t \right)} \right]\, = \,{M_\vartheta }\left[ {x\left( t \right),y\left( t \right)} \right]$, where $M$ is the standard rotation matrix with angle $\vartheta \in \left[ {0,\,2\pi } \right]$).
• Vertical and horizontal flips in the $x - $ and $\,y - \,$ axes $,\,x{^{^{\prime}}}\left( t \right) = \, - x\left( t \right),\,\,y{^{^{\prime}}}\left( t \right) = \, - y\left( t \right)$.
• Truncation of coordinates; we select two random times ${t_1}$ and ${t_2}$ such that $0 \unicode{x2A7D} {t_1} \unicode{x2A7D} T - {t_{{\text{min}}}}$ and ${t_1} + { }{t_{{\text{min}}}} \unicode{x2A7D} { }{t_2} \unicode{x2A7D} T$ resulting in truncated coordinates $x\left( {t^{\prime}} \right) = x\left( {{t_1}:\,{t_2}} \right)$ and $y\left( {t^{\prime}} \right) = y\left( {{t_1}:{t_2}} \right)$ (${t_{{\text{min}}}} = 20$, $T$ is trajectory length).

The augmentations are applied independently with a 30% probability for each training instance. The remaining features are calculated based on the augmented coordinates.

### 2.5 Training

We train the models using a batch size of 32 for a maximum of 30 epochs. During training, 10% dropout is applied to the first two biLSTM cells. Training converges typically before this and the model with the smallest validation loss is saved. A scheduler is implemented, decreasing the learning rate by a factor of ten every five epochs, if there is no improvement in validation loss. We use the Adam optimiser with an initial learning rate of $1\, \times {10^{ - 3}}$ and a weight decay ($\lambda $) of $2\, \times {10^{ - 6}}$ to prevent overfitting [60].

The loss functions for a single output timeseries are given by:

\begin{equation*}{\mathcal{L}_\alpha } = \frac{1}{T}\mathop \sum \limits_{i = 1}^T \left| {{ }{\alpha _{{\text{p}},i{ }}} - { }{\alpha _{{\text{g}},i{ }}}} \right|\end{equation*}

\begin{equation*}{\mathcal{L}_K} = \frac{1}{T}\mathop \sum \limits_{i = 1}^T \left| {{\text{log}}\left( {{K_{{\text{p}},i{ }}} + 1} \right) - {\text{ log}}\left( {{K_{{\text{g}},i{ }}} + 1} \right)} \right|{ }\end{equation*}

\begin{equation*}{\mathcal{L}_s} = \frac{1}{T}\mathop \sum \limits_{i = 1}^T - {w_{{y_i}}}\log p\left({y_i}\right)\end{equation*}

where ${\alpha _{\text{p}}}$ and ${K_{{\text{p }}}}$ are the predicted timeseries, ${\alpha _{\text{g}}}$ and ${K_{{\text{g }}}}$ are the ground truth timeseries, and $T$ is the total length of the protein trajectory (${T_{{\text{max}}}} = { }200)$. The state labels are imbalanced so we include class weights, ${w_{{y_i}}}$ (inverse frequency from training dataset) in the state loss function $\left( {{\mathcal{L}_s}} \right)$. The losses are normalised by trajectory length to account for varying lengths. The backpropagated loss is given by the average over the batch size (B) plus a regularisation term $\overline {\cal L} = \frac{1}{B}\sum\nolimits_{i = 1}^B {{{\cal L}_i}} + {\mkern 1mu} \lambda \sum\nolimits_{j = 1}^n {\theta _j^2} {\mkern 1mu} $. As the range of $K$ is between 10^−12^ and 10^6^, we work in the ${\text{lo}}{{\text{g}}_{10}}\left( {K + 1} \right)$ space, in which the value of the variable ranges between 0 and 6. This provides a normalised range for the model. All postprocessing steps on the predicted timeseries for $K$ are performed in its ${\text{lo}}{{\text{g}}_{10}}\left( {K + 1} \right)$ space.

Training was performed on a single NVIDIA RTX A5000 GPU taking a maximum of 10 h for a single output variable. This can be done in parallel with multiple GPU instances. We had access to two GPUs, such that training took a total of 20 h.

### 2.6 Post-processing output

The output of the model is a timeseries of the same length as the input sequence. We smooth $K\left( t \right)$ and $\alpha \left( t \right)$ by first replacing sections in which the output series varies by $0.01$ with their mean. This removes any noisy variations in the output sequence and considers jumps in values that are above this threshold. After initial smoothing, we apply a median filter with a window size of 3. Median filters preserve edges well, providing better changepoint detection. As part of the challenge constraints, the minimum time spent in a state must be at least three frames; hence, the state series is smoothed such that any state segment lasting fewer than three consecutive frames is deemed invalid and replaced with the value of its preceding state.

### 2.7 Changepoint detection

Changepoint detection was done using the ruptures library in Python [47]. Ruptures implements offline detection methods using a variety of cost functions. As we do not know the number of changes beforehand, an additional penalty parameter is applied. To obtain an ideal penalty value, we performed a quick grid-search over a sensible range of starting values to maximise the Jaccard index (described shortly) across the training set. Specifically, we use the Pruned Exact Linear Time (PELT) algorithm with a Least Squares Deviation (L2) cost function and a penalty of 0.3 [61]. This identifies changepoints in a timeseries by segmenting data to minimize the sum of squared deviations within segments, plus a penalty term dependent on the number of segments. We set the minimum distance of changepoints to be at least three-time steps and we min-max normalise the predicted timeseries for $\alpha $ and $K$ before changepoint detection. For the state series, changepoint detection is easier as the label value changes.

Given changepoints from all three outputs, we combined them by adding changepoints from $\alpha $ and $s$ to the $K$ changepoints and removing duplicates within a window size of 5. This centres changepoints around the $K$ changepoints, where the model generally performed better on. We cannot only take $K$ changepoints, as $K$ could be constant across a changepoint while $\alpha $ may not be, leading to missing changepoints.

### 2.8 Evaluation metrics

To evaluate the model on unseen testing data we calculate the mean absolute error per timestep for continuous variables, given by:

\begin{equation*}\frac{1}{N}\,\mathop \sum \limits_{j = 1}^N \frac{1}{{{T_{j\,}}}}\mathop \sum \limits_{i = 1}^{{T_{j\,}}} \left| {\,{p_{i,j\,}} - \,{g_{i,j\,}}} \right|\end{equation*}

where $N$ is the number of trajectories, ${T_{j\,}}$ the number of timesteps for the ${ }j{\text{th}}$ trajectory, ${p_{i,j\,}}$ is the $i$th predicted value of $\alpha $ or ${\text{log}}\left( {K + 1} \right)$ for the $j$trajectory and ${g_{i,j\,}}$ is the ground truth for the corresponding predicted point. For $\alpha $, this is simply the mean absolute loss (MAE), whereas for $K$, this is the mean absolute log error (MALE), as we work in the ${\text{lo}}{{\text{g}}_{10}}\left( {K + 1} \right)$ space.

For the state variable, we evaluate the confusion matrix (row-wise normalised), as the class distributions are imbalanced, and the state loss (${\mathcal{L}_{\text{s}}})$ averaged over all trajectories. Evaluation of changepoint detection is done using two indicators discussed below: the Jaccard index and the root mean square error (RMSE).

The Jaccard index measures the level of overlap between the predicted changepoints and the ground truth changepoints with an allowable error of 5 timesteps, in-line with the challenge constraints. For two sets A and B, the Jaccard index is defined by:

\begin{equation*}J = { }\frac{{A{{\mathop \cap \nolimits}}B}}{{A{{\mathop \cup \nolimits}}B}} = { }\frac{{{\text{TP}}}}{{{\text{TP}} + {\text{FN}} + {\text{FP}}}}{ }\end{equation*}

where ${\text{TP}}$, ${\text{FN}}$, and ${\text{FP}}$ are the number true positives, false negatives, and false positives respectively.

The RMSE for a single trajectory is given by [46]:

\begin{equation*}{\text{RMSE}} = { }\sqrt {\frac{1}{{{N_{{\text{CP}}}}}}{ }\sum\limits_{{\text{paired CPs}}} {{\left( {{t_{g,i}} - {t_{p,j}}} \right)}^2}{ }} \end{equation*}

where ${N_{{\text{CP}}}}$ is the number of changepoints in the trajectory, ${t_{{\text{g}},i}}$ is the ground truth changepoint, and ${t_{{\text{p}},j}}$ is the predicted changepoint for some pair of changepoints $\left( {i,j} \right)$. The RMSE is only calculated for paired changepoints ($ &lt; \!\!5$ timesteps away). Matching predicted and ground truth changepoints is performed using the Hungarian algorithm, performing cost minimisation, which is handled by the AnDi package [21, 46, 49].

## 3 Results

Work in single molecule imaging has primarily relied on classical statistics to calculate a single diffusion coefficient for an entire trajectory [13, 24–26]. These methods are difficult to implement, rely on expert input, and encounter large difficulties in detecting complex biological phenomena. With the rise of machine learning in the single molecule field, new models allow for the inference of diffusion properties at per timestep resolutions [21, 46]. Machine learning, particularly deep learning, removes the need for expert input and finetuned manual thresholding, while being able to detect complex states.

M3, an LSTM approach to the AnDi 2024 challenge [46], returns the diffusion coefficient, anomalous exponent, and protein state at every timestep of a protein trajectory, with an unknown number of changepoints. M3 is trained on simulations from biologically relevant scenarios including free and directed diffusion, confinement, and trapped motion.

Given a trajectory, the model extracts ten features from the coordinates, resulting in a multivariate timeseries. These features were selected after an extensive literature search [21, 29–46, 51–59], performing Pearson’s rank to remove correlated features, and greedy forward feature selection on the remaining features. The model is duplicated three times and trained independently for each output variable, with the exception of a LogSoftmax layer for the state variable as it is a multi-class variable.

During training, we implement augmentations; Gaussian noise, coordinate rotations, flips in the $x/y$ axes, and truncations of the time series (see *Methods*).

Once each model is trained, we perform inference on unseen trajectories from the test data set. Inference is performed once for each model, returning $\alpha \left( t \right)$, $K\left( t \right)$, and $s\left( t \right)$ for every timestep of the trajectory. Details of the dataset and simulation are described in table 1. The output timeseries have sudden changes reflecting changepoints in the protein trajectory.

M3 finished in the Top 5 of the AnDi 2024 challenge at the time of challenge closure [62]. Since then, we have fixed a bug in the training phase and slightly altered the model architecture to include another normalisation layer.

### **Pointwise estimation of** $\alpha $**, *K*, and *s***

3.1

We evaluate M3 on simulated trajectories from five underlying biological behaviours (see figure 1(a)) representing fBM with piecewise constant values of $\alpha \,\in \,\left[ {0,2} \right]$, $K\,\in $ [${10^{ - 12}},{ }{10^6}]{ }\left( {{\text{pixel}}{{\text{s}}^2}/{\text{fram}}{{\text{e}}^\alpha }} \right),{ }$ and state $s\,\in \,\left\{ {0,1,2,3} \right\}$. The protein states represent immobile (imm.) motion ($s = 0$), confinement (conf., $s = 1$), free diffusion ($s = 2$), and directed (dir.) motion ($s = 3$, $\alpha \, &gt; = 1.9$). Details of the simulation can be found in *Methods* *2.2*.

Once training is completed, we first visualise model performance by inspecting predicted outputs compared to ground truth variables for different trajectories from the test set. Hereof, we show all plots for $K$ in the ${\text{log}}\left( {K + 1} \right)$ space ($K \to {\text{ log}}\left( {K + 1} \right)$) such that all results are between $0\,$ and $6$. This is also evident from the scale on the plots.

**Figure 2.**
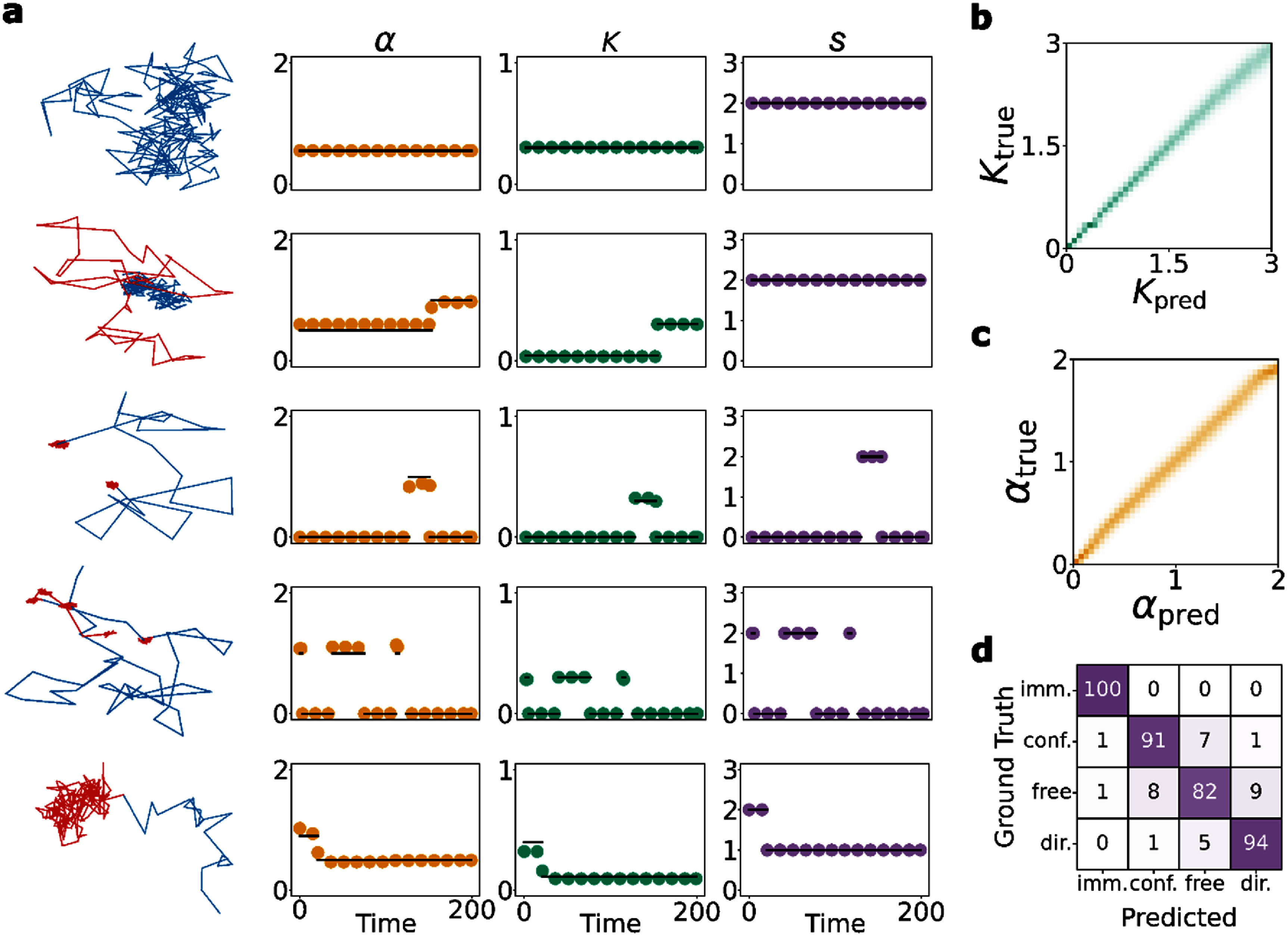
(a) Example model predictions on trajectories from the test set. We have (from top to bottom row), a single state, multi-state, two immobile trajectories with differing changepoint numbers, and lastly a confined trajectory. For clarity, not every timepoint prediction is shown. (b) $K$ predictions (in log space) compared to the ground truth. Trajectories for this plot were generated using the single state model and $\alpha $ was fixed at one. For every $K$ ground truth bin, 1000 protein predictions were averaged. (c) The same plot as (b) except varying $\alpha $, while $K$ was fixed at one. (d) Confusion matrix, row wise normalised, for the state variable over the entire test set.

Figure 2(a) shows example trajectories from different biological behaviours and the resulting model output. Generally, it was observed that predicted $K$ values are closer to the ground truth than $\alpha $, and immobile states ($s = 0$) were easily detected by the model, likely due to their distinct difference from the other states. The difference in prediction of $\alpha $ and $K$ can also arise from the inherent difficulty of the problem. Varying $K$ directly affects the step size of the protein whereas $\alpha $ can be seen as a measure of ‘directionality’, making it a more difficult task for the model to learn.

To verify our observations, we first calculate the mean absolute errors as measures of accuracy for $\alpha $ and $K$ averaged over the test set. This gives a MAE of 0.16 for $\alpha $ and a MALE of 0.14 for $K$. We also evaluate model predictions on trajectories simulated solely from the SSM for a wide range of $\alpha $ and $K$ values. Heatmaps of predictions against ground truth are shown on figures 2(b) and (c), and allowed for model evaluation while holding the other variable fixed. At an ensemble level, the predictions from both continuous variables lie close to the ground truth with the exception that $K\,$ ranges from $0$ to $3$ in log space. The restricted range of $K$ reflects the fact that the protein leaves the field of view during the simulation for diffusion coefficients larger than this. However, the range includes a wide range of diffusion coefficients ($0 - 100{ }\mu {{\text{m}}^2}\,{{\text{s}}^{ - 1}})$ beyond most experimentally observed values [63].

Due to the nature of the challenge and simulations, the test set is imbalanced, with the free state ($s = 2$) observed more frequently. We implement class weights (inverse frequency) during training and evaluate the confusion matrix. This gives an average class accuracy of $92\% $, as shown on figure 2(d). The confusion matrix verifies the ability of the model to detect immobile states ($s = 0$) very well compared to the other states. It is interesting to note the interplay between the states $s = 1$, $2$, and $3$. For certain trajectories, these three states can be confused for another, not necessarily due to an inherent fault in the model setup, but due to the difficulty of the classification. As an example, a slightly larger compartment ($s = 1$) can be detected as free diffusion ($s = 2$) and vice versa. As shown later, these effects can be amplified for shorter trajectories, where the model error increases, due to less information being available to the model. Another factor affecting this may stem from the use of class weights which favours minority classes, decreasing the accuracy of the model on the majority class.

### 3.2 Dependence on trajectory length

**Figure 3.**
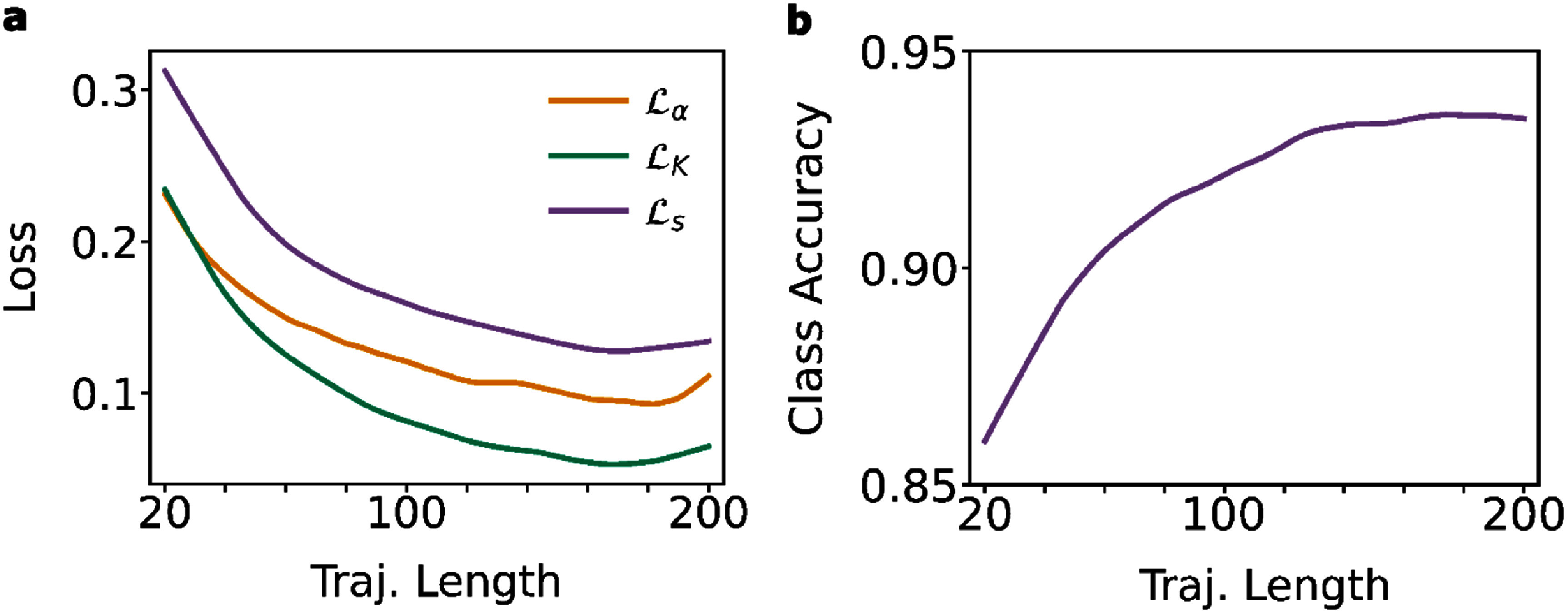
(a) Losses per timestep for each target variable with increasing trajectory length, averaged over the test set. (b) Average class accuracy with trajectory length over the test set.

We further evaluate model predictions over trajectory lengths in the range of $20$ to $200$. For this, we plot the model loss and average accuracy per timepoint for varying lengths across the test set. Consistent with previous work in the field, we a see a decrease in error as the trajectory length increases (see figures 3(a) and (b)). As more information is available to the model, better predictions are obtained. Shorter trajectories can originate from a range of possible parameters, leading to higher errors in $\alpha $, $K$, and $s$. Figures 3(a) and (b) show plateaus in both loss and class accuracy at lengths of roughly $150$ time steps and greater.

### 3.3 Changepoint detection

**Figure 4.**
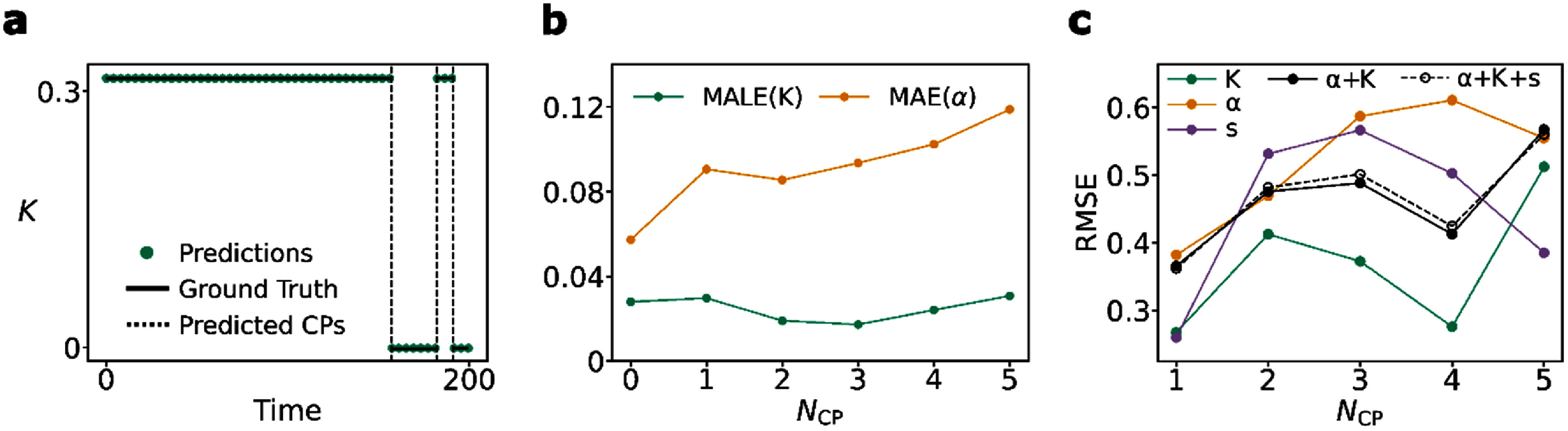
(a) Example application of changepoint detection, using the PELT algorithm [47], on the model prediction for $K$ (b) The MAE and MALE over the test set for varying number of changepoints (${N_{{\text{CP}}}}$) (c) RMSE for changepoint detection performed using different combinations of $\alpha $, $K$ and $s$ over the test set. Every ${N_{{\text{CP}}}}$ represents RMSE values for 100 randomly sampled protein trajectories of length 200 from the test set.

M3 does not directly return changepoints. Detecting changepoints in the output timeseries is performed using ruptures, an open-source changepoint detection library in Python, through a cost function and penalty parameter [47] (selected from a grid search across the training set, see *Methods* *2.7*). For the state timeseries, finding changepoints is easier as the value of the label changes. Figure 4(a) shows an example model prediction for $K$ and the detected changepoints. The model is able to pick up all changes in the protein trajectory for sufficiently long enough timeseries. The penalty parameter in ruptures is tuneable. To avoid constant tuning, all timeseries are min-max normalised before changepoint detection, resulting in the same penalty for both $K$ and $\alpha $.

As shown on figure 4(b), with an increasing number of changepoints (${N_{{\text{CP}}}}$) in the protein trajectory, the MAE for $\alpha $ increases while the MALE for $K$ remains roughly constant. This aligns with the asymptotic behaviour of $\alpha $, which leads to greater prediction estimates for a higher number of changepoints, in which the average segment length is shorter (inversely related) [21]. $K$ can be estimated with lower error from shorter segments (with less information per segment) and thus we see a constant loss as the number of changepoints increases.

Changepoint detection can be performed on the $\alpha $, $K$, and $s$ predictions. We first perform this individually, then combine sets of changepoints into a single set with repeats removed. This merges the changepoints from all timeseries and ensures the model is able to pick up on changepoints from any of the output variables. In figure 4(c), we evaluate the RMSE for changepoint detection using combinations of $\alpha $, $K$, and $s$. As expected, the $\alpha $ timeseries alone returns the highest RMSE across the range of changepoints, with changepoints from $K$ predictions providing the lowest RMSE values. The state timeseries alone is not enough to detect changepoints, since in multi-state trajectories, the state label remains the same although a changepoint can occur. However, using only $K$ timeseries for changepoints may mean we lose out on changepoints in which $K$ remains constant across the changepoint whereas $\alpha $ does not. Thus, we settle for using $\alpha + K$ changepoints (see figure 4(c)), while removing duplicates, for producing a set of changepoints for any given trajectory (providing slightly lower RMSE values than $\alpha + K + s$). This may mean we miss on changepoints in which both of these variables do not change, while $s$ does, but these trajectories are less likely ($\sim $0.04% of test dataset). Typically, a change in biological behaviour is accompanied by a change in diffusion coefficient or anomalous exponent.

**Figure 5.**
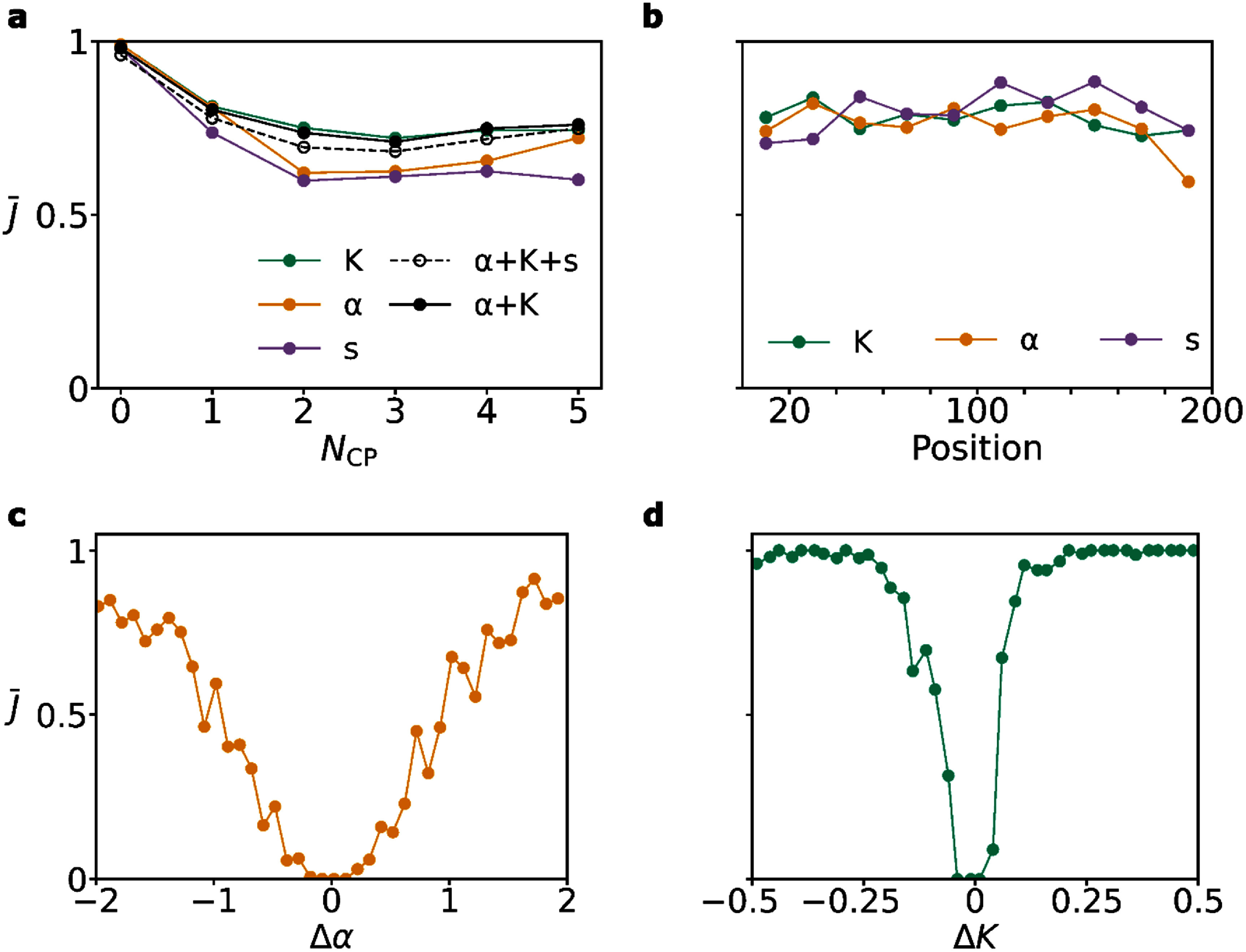
(a) Average Jaccard index, $\bar J,$ using combinations of variables for changepoint detection. Each point represents an average over 100 randomly selected protein trajectories of length 200 from the test set. (b) Average Jaccard index for trajectories of length 200 from the test set with a single changepoint. Each point represents the average over 100 randomly selected trajectories and bins a range of 20 lengths. (c) Average Jaccard index for trajectories with a single changepoint (thus having two segments, multi-state model), with $\Delta \alpha $ being the difference in $\alpha $ between the two segments. $K$ is fixed at one for both segments. Each point is the average of roughly 50 trajectories of length 200. (d) The same plot as (c), except for $\Delta K$, with $\alpha $ fixed at one between segments.

We also evaluate the average Jaccard index on figure 5(a) for varying changepoints using the same sets of variables. Again, we see slightly better performance across the test set using $\alpha + K$ compared to $\alpha + K + s$. Figure 5(b) shows changepoint detection for $\alpha $, $K$, and $s$ for trajectories with a single changepoint across the test set. The model’s bidirectional nature allows for consistent changepoint detection across various changepoint positions in trajectories.

Changepoint detection is clearly dependent on the difference of $\alpha $ and $K$ at a changepoint. Little to no difference in $\alpha $ or $K$ at a transition in a protein trajectory leads to no changepoint being detected using the PELT algorithm. For small differences in diffusive properties at a changepoint, the model may also return a single value at all timesteps for the entire trajectory, as it is unable to detect a change.

To evaluate performance as a function of the difference at a changepoint, we simulated two new datasets in which there exists a single changepoint exactly halfway through the trajectory (MSm), with all trajectories of length 200. The first dataset fixes $\alpha $ at one for all trajectories, allowing $K$ to change between the two segments, while the second dataset fixes $K$ at one for all trajectories and allows $\alpha $ to vary between the two segments. These datasets allow for robust evaluation of changepoint detection for varying $\Delta \alpha $ and $\Delta K$ between the segments. We show the Jaccard index for $\Delta \alpha $ between segments on figure 5(c). The model performs well for large differences in $\Delta \alpha $ between the segments, with the Jaccard index drastically reducing for smaller differences. As expected, we obtain a Jaccard value of zero when the two segments have identical $\alpha $ values, as the model did not pick up any changepoints. Figure 5(d) repeats the same analysis for $\Delta K$. This shows high values of Jaccard index for a much larger range of values, again reducing for $\Delta K$ close to zero. As mentioned before, we believe the increased performance in $\Delta K$ is due to a change in $K$ directly affecting the trajectory step sizes. Trajectories with fixed $K$ and increasingly closer values of $\alpha $ are much harder to distinguish as the model needs to spot a measure of ‘directionality’ over stochastic consecutive time steps.

### 3.4 Dependence on biological behaviours

**Figure 6.**
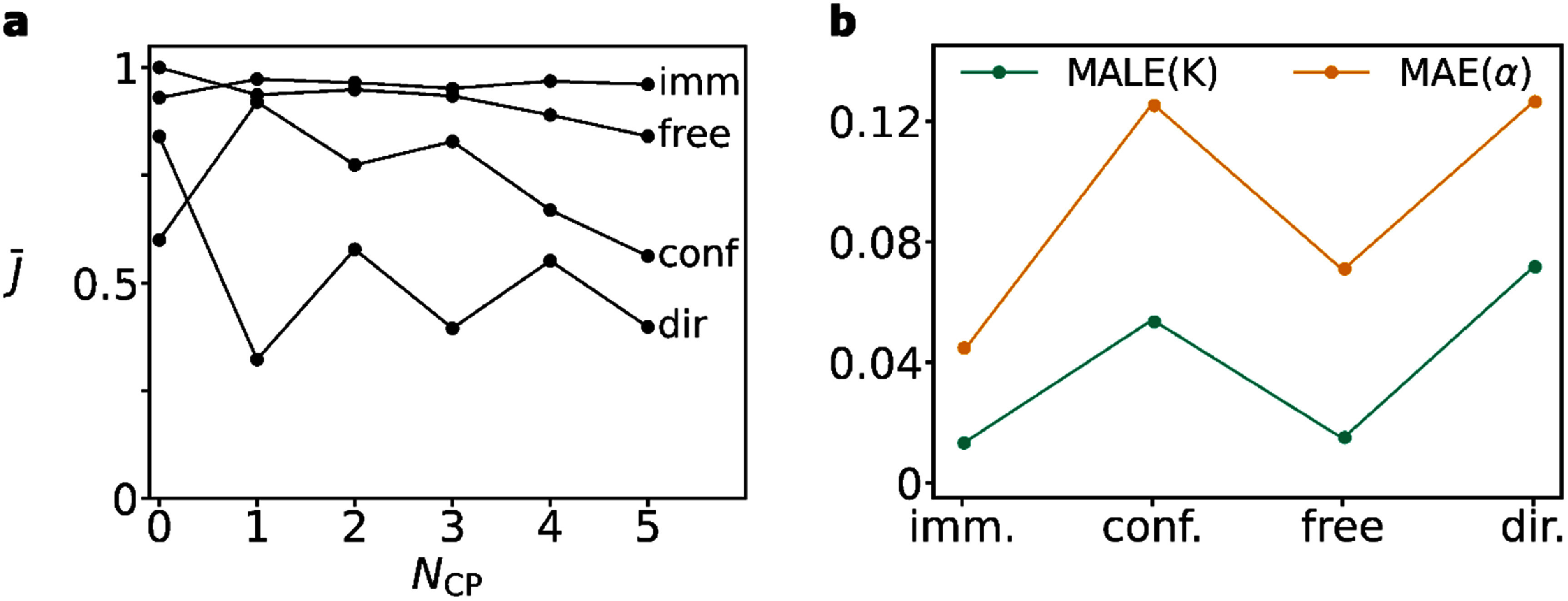
(a) Average Jaccard index for changepoint detection for various biological states and number of changepoints ${N_{{\text{CP}}}}$. Each point represents the average over 100 randomly selected protein trajectories of length 200. (b) MAE($\alpha $) and MALE($K$) given the ground truth biological state over the entire test set.

Lastly, we evaluate the Jaccard index using the combined changepoints from $\alpha + K$ timeseries against simulated biological states. As figure 6(a) highlights, the Jaccard index decreases as the number of changepoints increases for most biological states. The immobile and free states provide high Jaccard values over a range of changepoints. We see decreased detection of changepoints using the $\alpha + K$ set for the confined and directed models (any trajectories containing $s = 1$ and $s = 3$ respectively), particularly for a greater number of changepoints. This aligns with figure 6(b), which shows significantly higher values of MAE and MALE for the confined and directed models. The larger error in $\alpha $ and $K$ for these models directly affects subsequent changepoint detection, so we expect to see decreases Jaccard indices. The higher error in the confined model may arise from large values of $K$ we simulated. As the protein reflects at a compartment boundary, consecutive timesteps can appear close for both high and small values of $K$. This may impact the model negatively, as it sees higher and lower values of $K$ during training for similarly looking coordinates. A similar issue may be present for directed trajectories, where high values of $K$ can lead to large displacements, potentially creating trajectories similar to lower $K$ values and higher values of $\alpha $.

## 4 Discussion

In this work we have presented M3, a machine learning model with three stacked biLSTMs layers for inferring diffusion coefficients, anomalous exponents, and states, for every frame of a protein trajectory. Specifically, we show M3 performs well on noisy stochastic trajectories simulated from varying biologically relevant scenarios.

M3 provides a new and simple method for detecting complex motion of proteins in cells. Additionally, the model does not require prior expert knowledge or manual finetuning of thresholds, as done with classical statistics or methods relying on MSD. In the AnDi Challenge, at the time of challenge closure, the model placed in the Top 5 on a set of combined metrics including absolute errors, Jaccard indices, and RMSE of predicted changepoints [62]. Since then, improvements have been made to the model, predominantly in the model architecture (addition of another layer normalisation) and the correction of a bug in the training data generation. The model consists of roughly half a million trainable parameters per variable, making it possible to train on a single NVIDIA GPU in 10 h for a single output variable.

It is possible to combine the models into a single model with three output heads, but this is difficult as it combines classification and regression. This can serve as future work, as a more complex loss function with additional parameters would be required.

Applying M3 to experimental single-molecule data requires consideration of several caveats commonly encountered in single molecule imaging. Fluorophore blinking may lead to intermittent signal loss. This can be mitigated through longer exposure times which increase photon collection and reduce blinking effects although at the cost of temporal resolution. The use of more photostable fluorophores will reduce both blinking and photobleaching enabling longer trajectory lengths. Excitation laser intensities in single molecule experiments can also be tuned to access longer timescales, which would be beneficial when wanting to observe various diffusive behaviours and allows for best model performance. Lower laser intensities also reduce potential photo-induced damage to living cells.

In cases where missing frames arise due to blinking, we can interpolate coordinates from surrounding frames. As part of this process accurate localisation is critical. Out-of-focus molecules, reduced signal-to-noise, and motion blur can introduce localisation errors leading to errors in downstream diffusion parameters. A potential approach to tackling localisation errors would be to resample trajectory coordinates by adding Gaussian noise based on localisation uncertainty. Subsequently, inference on each trajectory can be used to plot a probability distribution over possible output values. The output distribution can be used to infer both a final value and error range (similar to MC dropout techniques). Such an approach will be useful in cases where localisation cannot be performed accurately and provides pointwise errors.

Lastly, specific cellular boundaries will constrain diffusion. As such, it may be better to finetune the model using simulated tracks that account for cell geometry. Finetuning ML models on experimental data will be harder and subjective as compared to simulated data due to small dataset sizes and the lack of a ground truth.

As a starting point for experimental validation, future work can involve the use of M3, without any further finetuning, to analyse binding kinetics. M3 performs incredibly well (see figure 2(d)) in detecting immobile states which can be used to calculate dwell times, crucial for understanding cellular mechanisms and gene expression *in vivo* [16–18].

Improvements can be also made to the model architecture, such as the addition of convolutional layers, which would require further training and hyperparameter optimisation. Convolutional layers may be less flexible with varying length timeseries but can be incorporated in a unified (multi-headed) architecture. The model can also be entirely replaced by transformers, which have shown to outperform LSTMs. Transformers alongside hybrid LSTM-CNN models were tested. All architectures were kept small (minimising free parameters) for compatibility with single-GPU training within a day, but none outperformed the evaluation metrics as given by the current M3 model. However, experimentation was brief and should be further explored.

Future changes to the model architecture should potentially focus on short trajectories ($ &lt; \!\!150$ timesteps) as this is where the model performs worse, with a plateau in accuracy for longer trajectories ($ &gt; \!\!150$ timesteps). For current applications to experimental single molecule data, longer trajectories are preferable, which may require running experiments using low laser intensities and photostable fluorophores.

The ability of machine learning methods to achieve better performance, as shown by the AnDi challenges, while also providing pointwise diffusive properties, highlights the need for a shift in analysis of experimental data [21, 46]. New techniques, such as M3, provide fine grained insights into diffusive behaviours, allowing for better understanding of cellular mechanisms. Combined with advancements in experimental data and techniques, this can lead to new discoveries in protein behaviour and mechanisms at cellular levels.

## Data Availability

The data that support the findings of this study are openly available at the following URL/DOI: https://github.com/raschedh/AnomalousDiffusion.
